# Needling therapy for myofascial pain: recommended technique with multiple rapid needle insertion

**DOI:** 10.7603/s40681-014-0013-2

**Published:** 2015-02-03

**Authors:** Li-Wei Chou, Yueh-Ling Hsieh, Ta-Shen Kuan, Chang-Zern Hong

**Affiliations:** 1 Department of Physical Medicine and Rehabilitation, China Medical University Hospital, Taichung, Taiwan; 2 School of Chinese Medicine, College of Chinese Medicine, China Medical University, Taichung, Taiwan; 3 Research Center for Chinese Medicine & Acupuncture, China Medical University Hospital, Taichung, Taiwan; 4 Department of Physical Therapy, Graduate Institute of Rehabilitation Science, China Medical University, Taichung, Taiwan; 5 Department of Physical Medicine and Rehabilitation, National Cheng Kung University Hospital, Tainan, Taiwan; 6 College of Medicine, National Cheng Kung University, Tainan, Taiwan; 7 Department of Physical Therapy, Hung Kuang University, Chung-Chie Road, Taichung, Taiwan

**Keywords:** Acupuncture, Analgesia, Mechanism, Myofascial trigger point, Needling

## Abstract

Myofascial trigger point (MTrP) is a major cause of muscle pain, characterized with a hyperirritable spot due to accumulation of sensitized nociceptors in skeletal muscle fibers. Many needling therapy techniques for MTrP inactivation exist. Based on prior human and animal studies, multiple insertions can almost completely eliminate the MTrP pain forthwith. It is an attempt to stimulate many sensitive loci (nociceptors) in the MTrP region to induce sharp pain, referred pain or local twitch response. Suggested mechanisms of needling analgesia include effects related to immune, hormonal or nervous system. Compared to slow-acting biochemical effects involving immune or hormonal system, neurological effects can act faster to provide immediate and complete pain relief. Most likely mechanism of multiple needle insertion therapy for MTrP inactivation is to encounter sensitive nociceptors with the high-pressure stimulation of a sharp needle tip to activate a descending pain inhibitory system. This technique is strongly recommended for myofascial pain therapy in order to resume patient’s normal life rapidly, thus saving medical and social resources.

## 1. Introduction

### 1.1. Background of Myofascial trigger point (MTrP)

Originally, myofascial trigger point (MTP) was defined by Travell and Simons [1, 2] as most tender (hyperirritable) spot in a palpable taut band of skeletal muscle fibers and basic cause of myofascial pain syndrome. They also defined latent MTrP is tender, but not painful spontaneously; active MTrP is painful spontaneously or in response to movement of the involved muscle. Almost all adults have latent MTrPs in most skeletal muscles, but no latent MTrPs in children under the age of one year [3]. Latent MTrPs may develop after age one or later when children grow up with repetitive minor traumas to nerves or muscles [4]. Pressure stimulation of an MTrP can elicit pain, referred pain, and local twitch response (LTR) (brisk contraction of muscle fibers in its taut band), all characteristics of an MTrP. [1, 2] Pain elicited by compression of this spot is familiar to the patient as the usual pain complaint (pain recognition) [2]. It has been suggested that “spot tenderness”, “taut band”, and “pain recognition” are crucial for diagnosis; “referred pain” and “local twitch responses” serve as “confirmatory signs” for MTrP diagnosis [5]. Clinically, myofascial pain syndrome includes any phenomenon due to activation of latent MTrPs as a consequence of a certain pathological conditions: e.g., chronic repetitive minor muscle strain, poor posture, systemic disease, neuromusculoskeletal lesions (sprain, strain, bursitis, enthesopathy, arthritis, vertebra disc lesion) [6-8]. In clinical observation, if an MTrP is not appropriately treated and associated underlying pathological lesion not eliminated, it can be expanded to other regions and develop other active MTrPs. [2, 6, 8-11] Original MTrP is called primary or key MTrP, later developed ones are secondary or satellite MTrPs [1]. Inactivation of a key MTrP can subsequently eliminate satellite MTrPs [2, 10].

### 1.2. Pathophysiology of myofascial trigger point

**Fig. 1:**
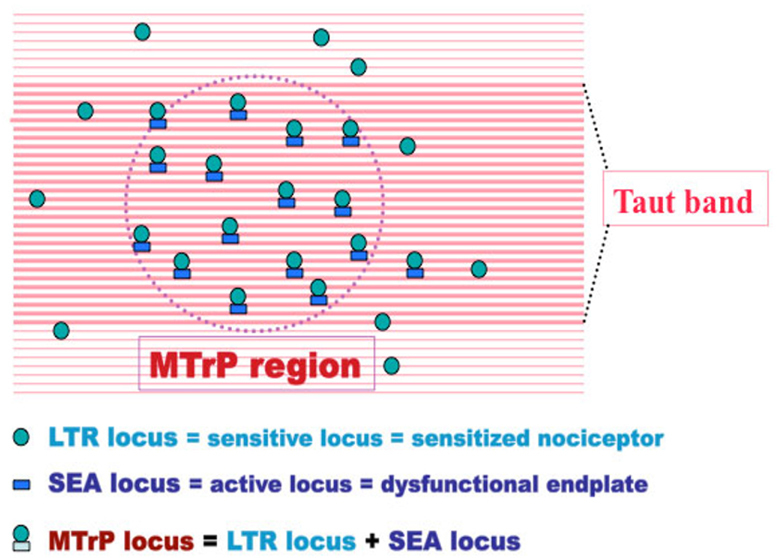
Multiple MTrP loci in a myofascial trigger point region.

**Fig. 2:**
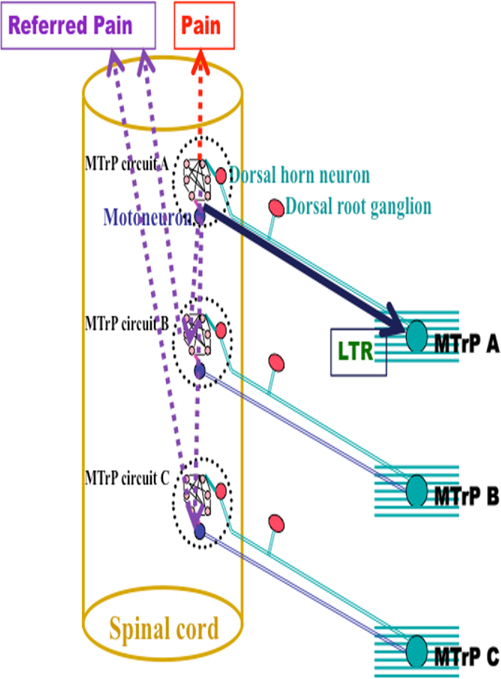
Connection of “myofascail trigger point circuit” (“MTrP circuit”) in the spinal cord.

**Fig. 3:**
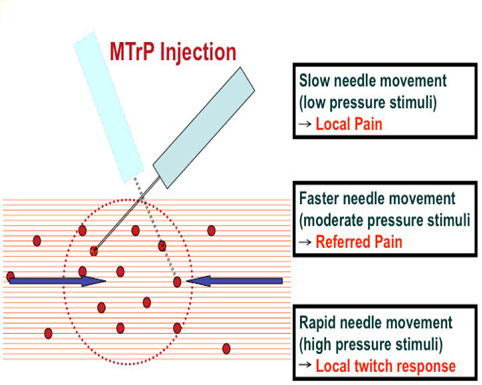
Stimulation of a sensitive locus with needle tip during MTrP injection to elicit pain, referred pain or local twitch response.

Recent studies on both human subjects and animals suggest multiple MTrP loci in an MTrP region [7, 10] and an MTrP locus containing a sensory (sensitive or LTR locus) and a motor component (active, spontaneous electrical activity, or SEA locus). (Figure 1) [7, 8, 10, 11]. Stimulation of a sensitive locus elicits local pain, referred pain, and local twitch response [7, 8, 11, 12]. Hong suggested that an MTrP is integrated in the spinal cord via a “myofascial trigger point circuit (MTrP circuit)” (Figure 2) [6, 8, 11]. Nociceptors in an MTrP region connect to a group of dorsal horn cells (sensory neurons) in the spinal cord, “MTrP related sensory neurons” responsible for central sensitization and transmission of pain information to the brain. The neural network with connections among them is defined as an “MTrP circuit” [6, 13]. Such a circuit corresponding to a certain MTrP can send nerve branches to connect with another MTrP circuit corresponding to other MTrPs. Latent MTrP may activate if stimuli from peripheral sites are strong enough to trigger its MTrP circuit. Mechanical stimulation to a sensitive locus may elicit local pain if strong enough. Stronger stimulation may elicit referred pain to a remote region. Very strong (such as a tiny needle tip) stimulation may elicit local twitch response (Figure 3). Histological study suggests sensitive locus as actually a free nerve ending, a sensitized nociceptor [7, 8, 11]: i.e. MTrP as a region accumulating multiple sensitized nociceptors [8, 11] whose irritation or sensitization of nociceptors causes spontaneous pain. Yet pain from stimulation of sensitized nociceptors differs from pain elicited by stimulation of normal (non-sensitized) nociceptors. In clinical practice (especially during MTrP injection), many patients distinguish these types of pain with different nature; they usually describe pain due to MTrP as a “sore pain” that occurs spontaneously (active MTrP) or is elicited by stimulation of sensitized nociceptors.

**Fig. 4:**
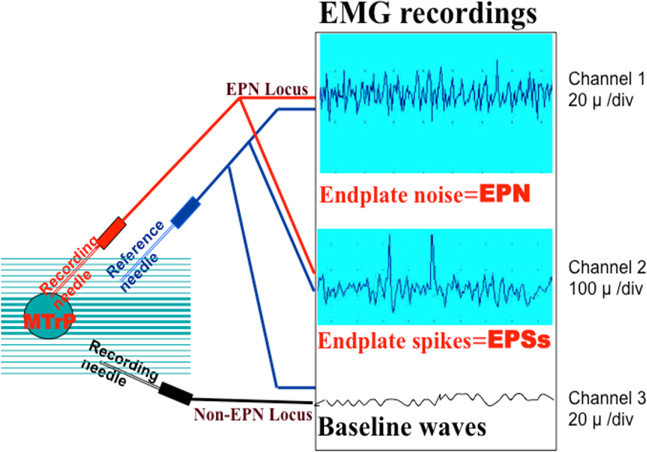
Spontaneous electrical activity (SEA) including endplate noise (EPN) and endplate spike (EPS) can be frequently recorded in a MTrP region.

**Fig. 5:**
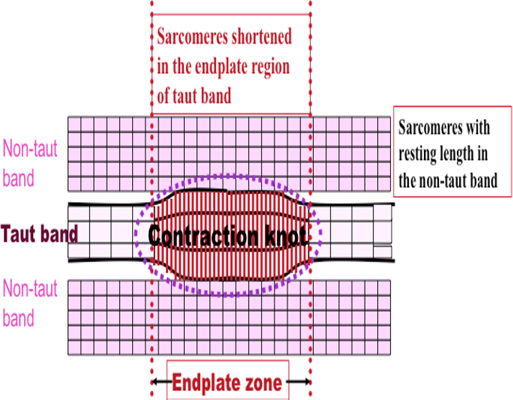
Contraction knot in the endplate zone of a taut band with shortening of sarcomeres, but relatively elongated sarcomeres outside the endplate zone, to increase tension of the taut band.

**Fig. 6:**
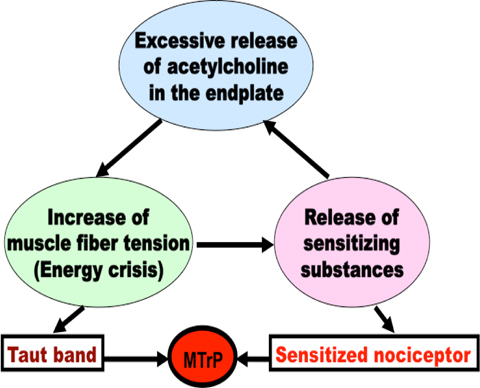
Simons’ integrated hypothesis of myofascial trigger point.

Spontaneous electrical activity, including endplate noise (EPN) and endplate spike (EPS), can be recorded from active (SEA or EPN) loci (Figure 4) [2, 7, 14-16]. Simons has strongly suggested MTrP always locating at an endplate zone of a muscle and EPN as always recorded from an MTrP region [17-22]; he has connected this finding to the formation of taut band [22]. In an early microscopic study, Simons et al. found contracture knots in the taut band of dog skeletal muscle fibers [23] (Figure 5). The EPN emanates from over leakage of acetylcholine (Ach) molecules in motor nerve endings, which cause shortening of sarcomere in the endplate zone only, but not extended to sarcomeres outside the endplate zone, since only graded (but not action) potentials has developed in this region. Sarcomere contraction can increase of tension in the muscle fibers (taut band). Due to the sarcomere contraction, the focal circulation is impaired but the energy requirement is increased, and thus cause energy crisis [22]. Simons has developed an “integrated hypothesis of MTrP” (Figure 6) [20, 22, 24, 25]. This integrated hypothesis has three essential features: excessive acetylcholine release, sarcomere shortening, and release of sensitizing substances [19]. Greater acetylcholine release aggravates muscle fiber tension (taut band) containing MTrP and subsequently causes “energy crisis” with increased metabolism, local ischemia and hypoxia that in turn induce secretion of sensitizing substances to cause pain. Sensitizing substances further cause abnormal acetylcholine release so that a vicious cycle is completed.

### 1.3. Treatment of Myofascial Trigger Point

In clinical practice, MTrPs due to over activity or inappropriate activity of muscle itself can be easily inactivated after appropriate rest and avoidance of overuse or inappropriate use. However, active MTrPs associated with pathological conditions including chronic repetitive minor muscle strain, poor posture, systemic diseases, or neuromusculoskeletal lesions (such as sprain, strain, bursitis, enthesopathy, arthritis, vertebra disc lesion, etc.) cannot be easily eliminated if the underlying or related lesion is not appropriately treated [2, 5-7, 10, 13]. If the underlying pathology is not appropriately and completely treated, the MTrP can only be inactivated temporarily, but never completely. However, in some situations, inactivation of MTrP is necessary. These conditions include unable to identify the underlying etiological lesion, difficulty in treating the underlying etiological lesion, intolerable pain prior to eliminate the etiological lesion, etc.

To inactive an MTrP, conservative treatment (such as appropriate systemic or local applied non-steroidal anti-inflammatory drug, thermotherapy, manual therapy, and other physical modalities) should be performed prior to more aggressive therapy (such as local steroid injection, spinal facet joint injection, MTrP injection, dry needling, or acupuncture), especially for acute lesions or mild lesions [2, 6, 7, 13, 26, 27].

## 2. Needling therapy for myofascial trigger points

Such therapy means any treatment with needles, including injection and dry needing. Injection entails introduction of drugs via an “injection needle” (containing a central hollow); dry needling involves penetration through skin without introducing any drug. Using a solid needle without central hollow or an injection needle with a central hollow can perform dry needling. MTrP injection with various solutions has been applied for inactivation of MTrPs: e.g., traditional MTrP injection with Travel’s technique [1], MTrP injection with botulinum toxin A [28-30], MTrP injection with multiple rapid insertions [9, 10, 31], injection of taut band plus MTrP [32], pre-injection blocks prior to MTrP injection [32].

MTrP is also inactivated by various techniques: traditional acupuncture [33-39], dry needling with EMG needle [31, 40-47], dry needling with electrical stimulation (similar to electrical acupuncture) [48-50], superficial dry needling [51-53] and Fu’s (remote) subcutaneous needling [54-57]. Kalichman and Vulfsons [58] suggested dry needling is a cheap, easy to learn with appropriate training, caring lower risk and minimally invasive treatment modality. With either MTrP injection or dry needling, MTrP itself can be needled directly or a remote site can be needled (remote needling therapy). In all cases, immediate and complete pain relief is most frequently obtained if multiple insertion technique is applied [1, 9, 10, 12, 40, 41, 47]. To date, we see this as the best technique of MTrP needling.

## 3. Mutiple needle insertion technique

### 3.1. Background of Multiple Needling Technique

**Fig. 7:**
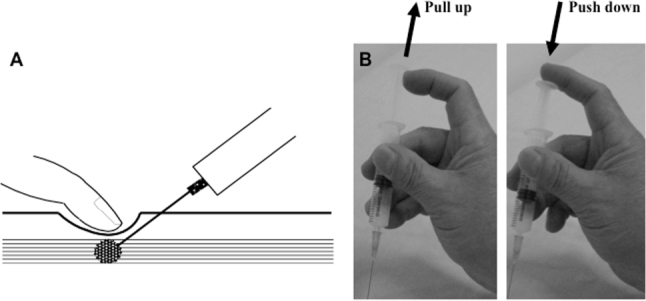
Hong’ rapid multiple needle insertion technique, including careful palpation of MTrP to direct the injection needle (A), and a special way of holding and controlling syringe with the palm firmly contact with patient’s body (B).

Traditional MTrP injection originally developed by Travell is actually multiple insertion [59]. The needle should be moved in-and-out into different directions to encounter sensitive spots in an MTrP region. In this way, MTrP pain can usually be almost completely eliminated immediately after most multiple sensitive spots are injected with a drop of local anesthetic agent on each site. Hong [9] has modified this technique to a fast-movement procedure in order to avoid tissue damage from side movement of needle or the grabbing of needle by an elicited LTR. Later, this new technique has been recommended by Simons [2] and widely use for trigger point injection or needling. Multiple rapid insertion technique modified by Hong includes a special way of holding a syringe and carefully palpating the taut band and tender spot (MTrP region) with a finger of non-dominant hand (not holding the syringe) (Figure 7). Palm of the hand holding a syringe must tightly contact patient’s body to avoid excessive penetration if the patient moves during injection. Careful palpation of the MTrP region reduces number of needle penetrations to avoid excessive bleeding or muscle fiber damage.

### 3.2. Modification of Multiple Rapid Needling Technique

Multiple needle insertion technique is widely accepted for MTrP inactivation, by either MTrP injection or dry needling [1, 9, 25, 42, 45, 46]. Acupuncture needle is smaller in size than regular injection needle and hence difficult to apply in MTrP region with rapid needle movement. Recently, Chou et al [40] developed a new technique of acupuncture therapy with simultaneous rotation of the needle to facilitate its in-and-out movement. This technique is similar to MTrP dry needling by insertion of acupuncture needle into multiple loci of MTrP regions with a fast insertion speed (to provide high-pressure stimulation to sensitive loci) to elicit LTRs easily. Simultaneous rotation of needle (fast screwed-in and screwed-out technique) expedites rapid needle movement and avoids bending of the small-sized acupuncture needle. This technique was originally developed to inactivate an MTrP in the upper trapezius by needling the MTrP at the ipsilateral forearm following the principle of acupuncture; it can also be applied in direct needling of an MTrP. When this techniques was developed, it was found that irritability (measured as subjective pain intensity, pain threshold, and amplitude change of EPN) of the MTrP in the upper trapezius muscle could be suppressed after needling remote acupoints [41]; this effectiveness was also confirmed by animal study [60, 61]. This technique is further recommended for myofascial pain therapy, a simple and rapid way to relieve chronic pain, using low-cost medical supplies. Once patients resume their normal lives, they behave much better to provide social contribution. Therefore, it is a cost-effective technique in medical care.

## 4. Proposed mechanism of multiple rapid needling therapy for pain control

Various theories explain possible mechanism [62]. Chinese traditional acupuncture for pain control is based on a Traditional Chinese Medicine (TCM) theory developed 2500 years ago [63, 64], but it lacks scientific proof. Many studies cite release of endogenous opiates in the central nervous system [65-74] after needling therapy; others suggested the existence of peripheral opiate receptors acting locally rather than systemically in needling analgesia via anti-inflammatory effect [75-77]. Serotoninergic descending pain inhibitory pathway for pain relief after needling therapy has also been recently studied [36, 72, 78-81]. Neural pathway for pain inhibition has been well accepted [36, 82-84]. Considering very fast response of pain suppression immediately after multiple rapid needle insertion, it is more likely that analgesic effect is via the nerve pathway rather the slow chemical reaction. Multiple mechanisms are very likely involved in needling analgesia [36], depending on type of needling.

## 5. Proposed mechanism of multiple rapid needling therapy

Purpose of multiple needle insertion during needling is to encounter as many sensitive loci in an MTrP region as possible; rapid needle movement leads high-pressure stimulation to sensitive loci to elicit more LTRs. As suggested by Hong [8, 85], the most likely mechanism of immediate and total pain relief after multiple and rapid needle stimulation is hyperstimulation analgesia [34] via descending pain inhibitory system. Strong pressure stimulation by rapid needle movement to the MTrP loci (sensitized nociceptors) can provide very strong neural impulses to dorsal horn cells in the spinal cord, breaking the vicious cycle of the “MTrP circuit” via descending pain inhibitory pathway [6, 13].

## 6. Conclusion

Best technique for total immediate inactivation of MTrP is “multiple rapid insertion.” It very likely provides high-pressure stimulation to the multiple sensitized nociceptors via the descending pain inhibitory pathway, quickly interrupting “MTrP circuit” vicious cycle to eliminate pain immediately. This technique is strongly recommended for myofascial pain in order to resume patient’s normal life rapidly, thus saving medical and social resources.

## Acknowledgement

This work was supported in part by Taiwan Ministry of Health and Welfare Clinical Trial and Research Center of Excellence (DOH102-TD-B-111-004) and by CMU under the Aim for Top University Plan of the Ministry of Education, Taiwan.
